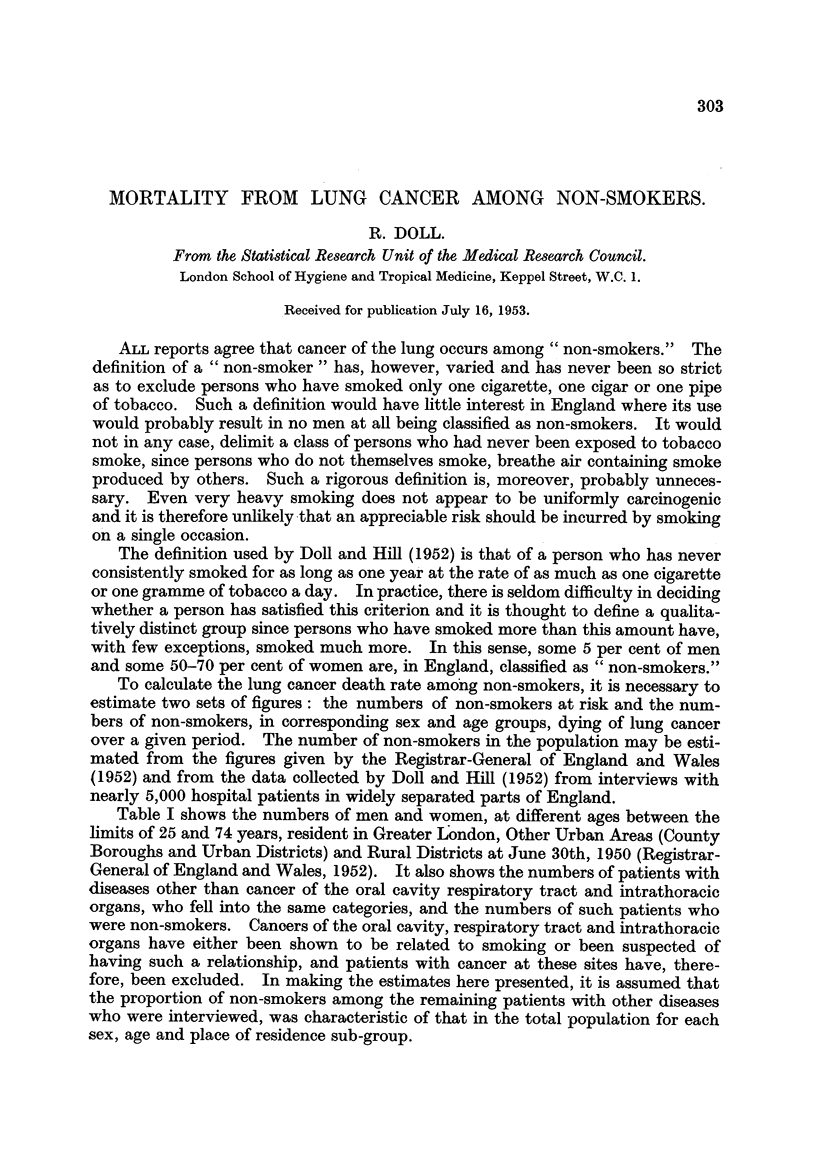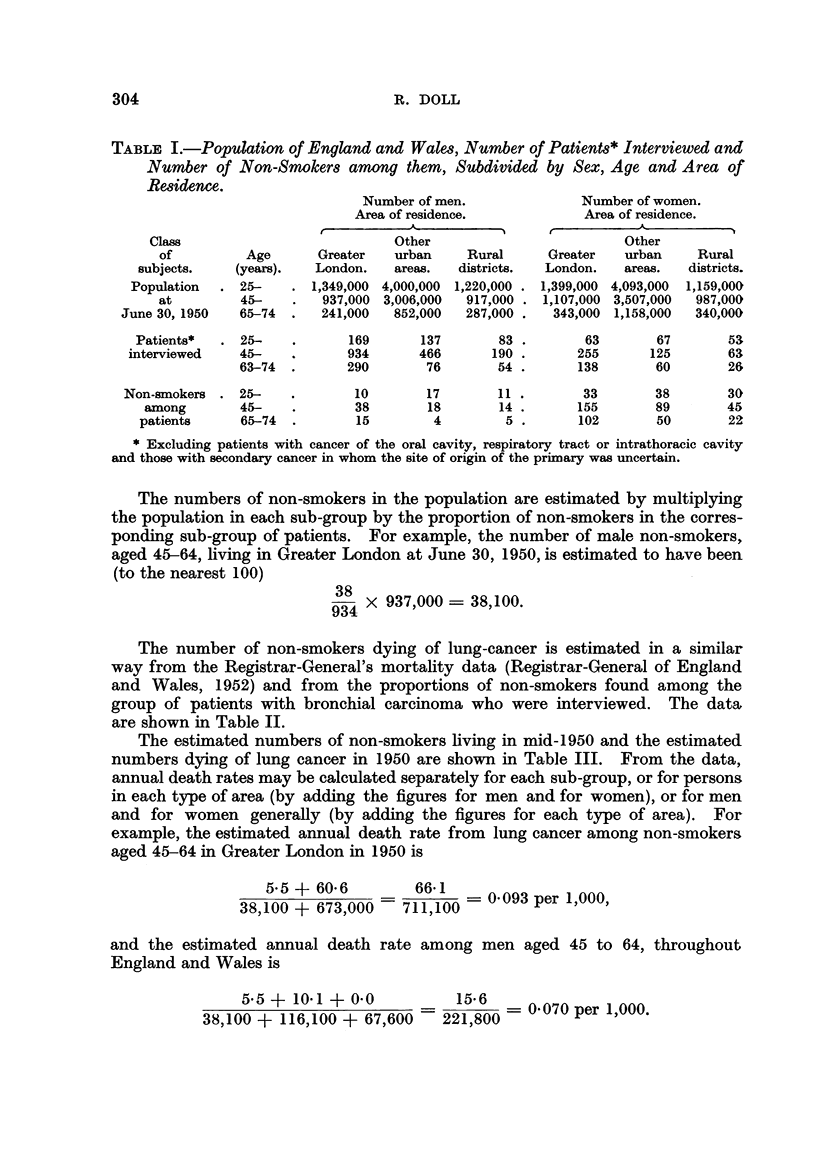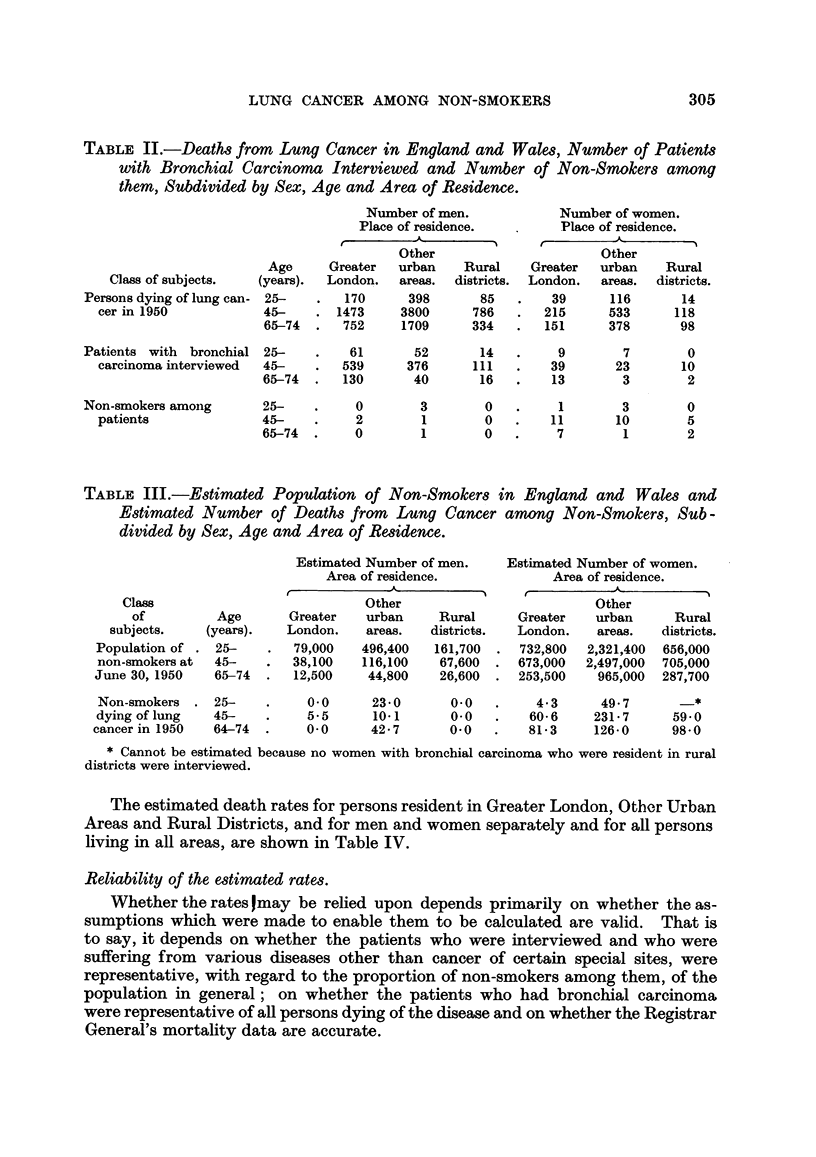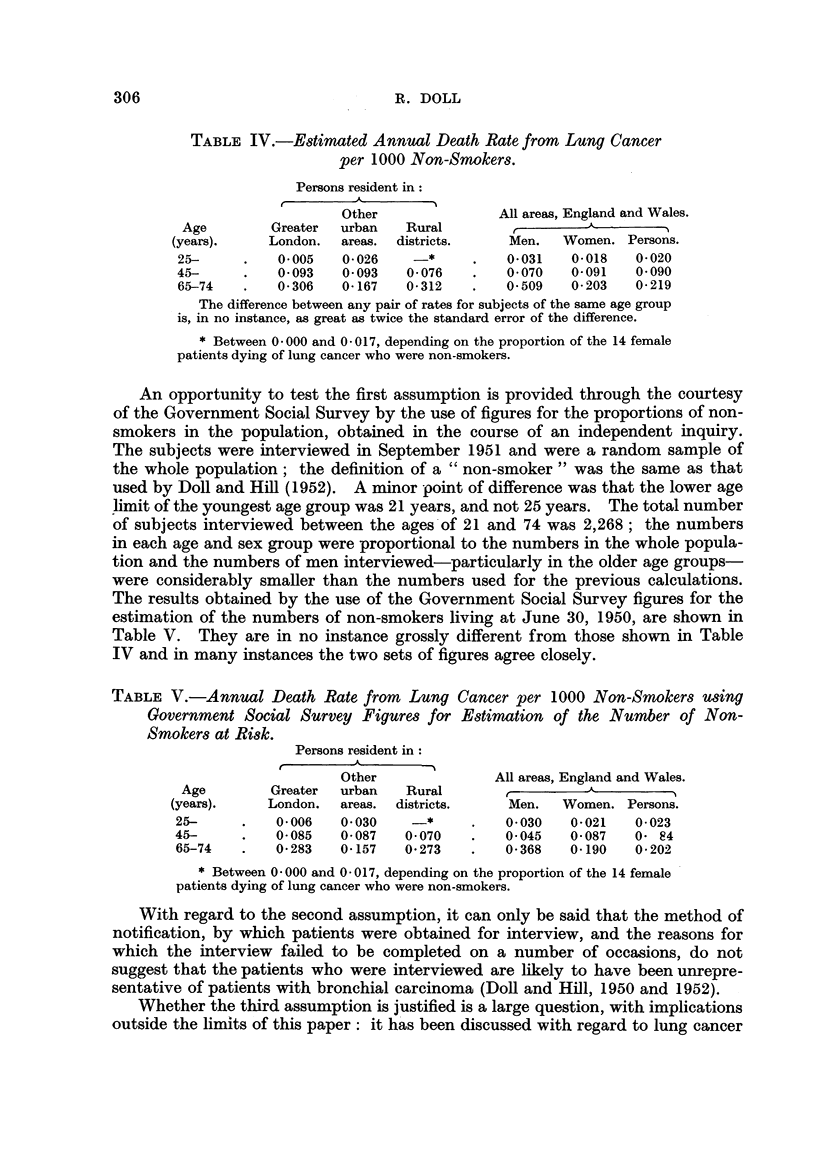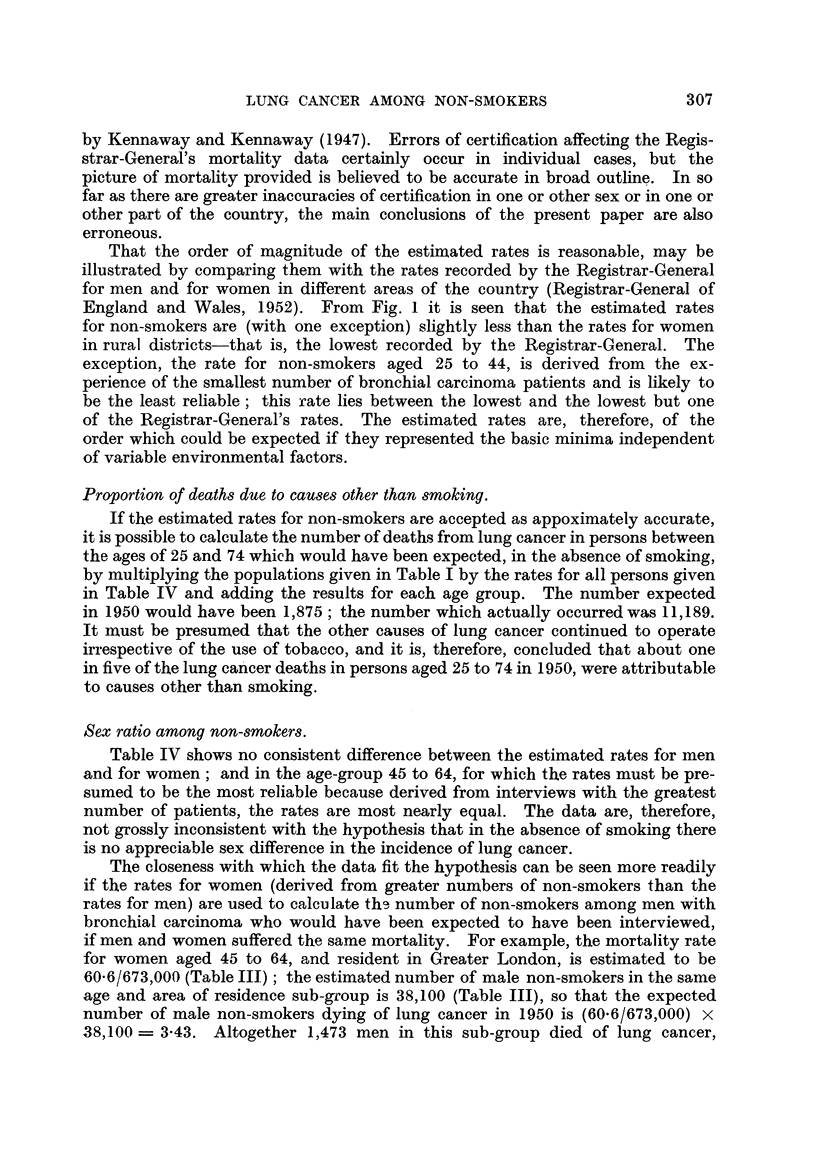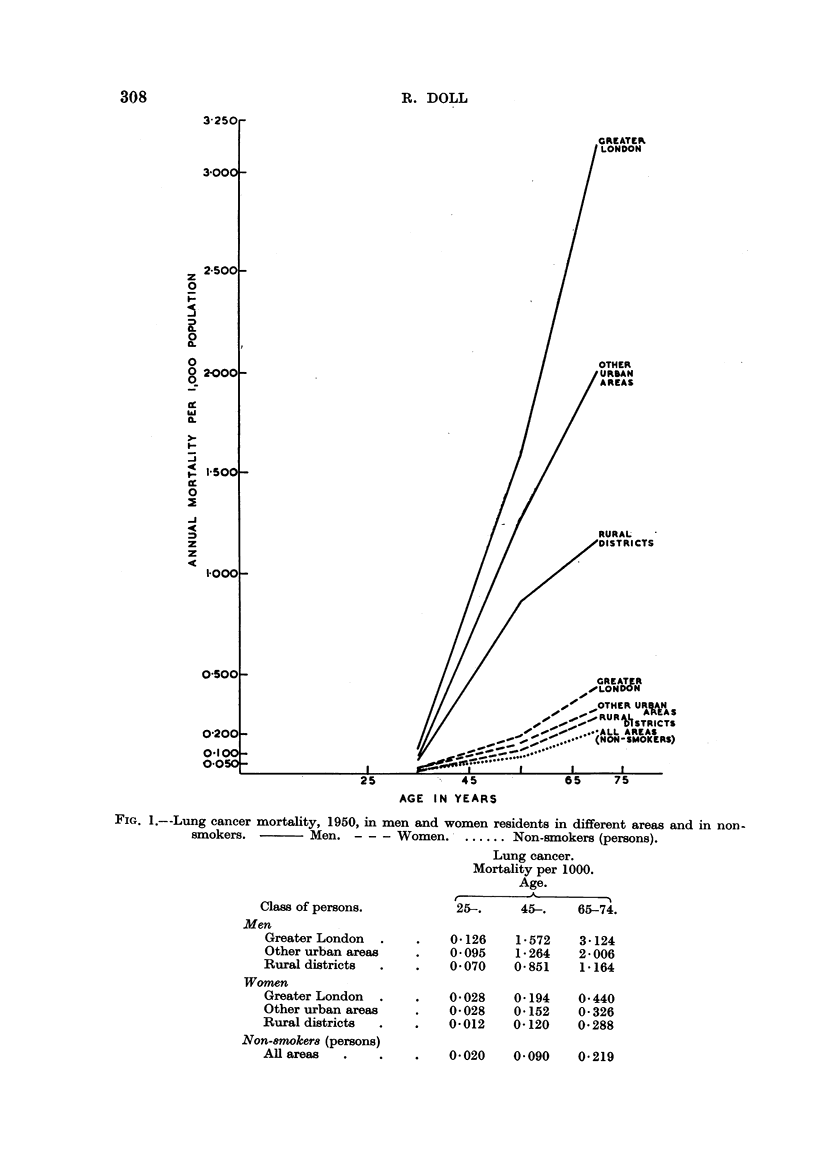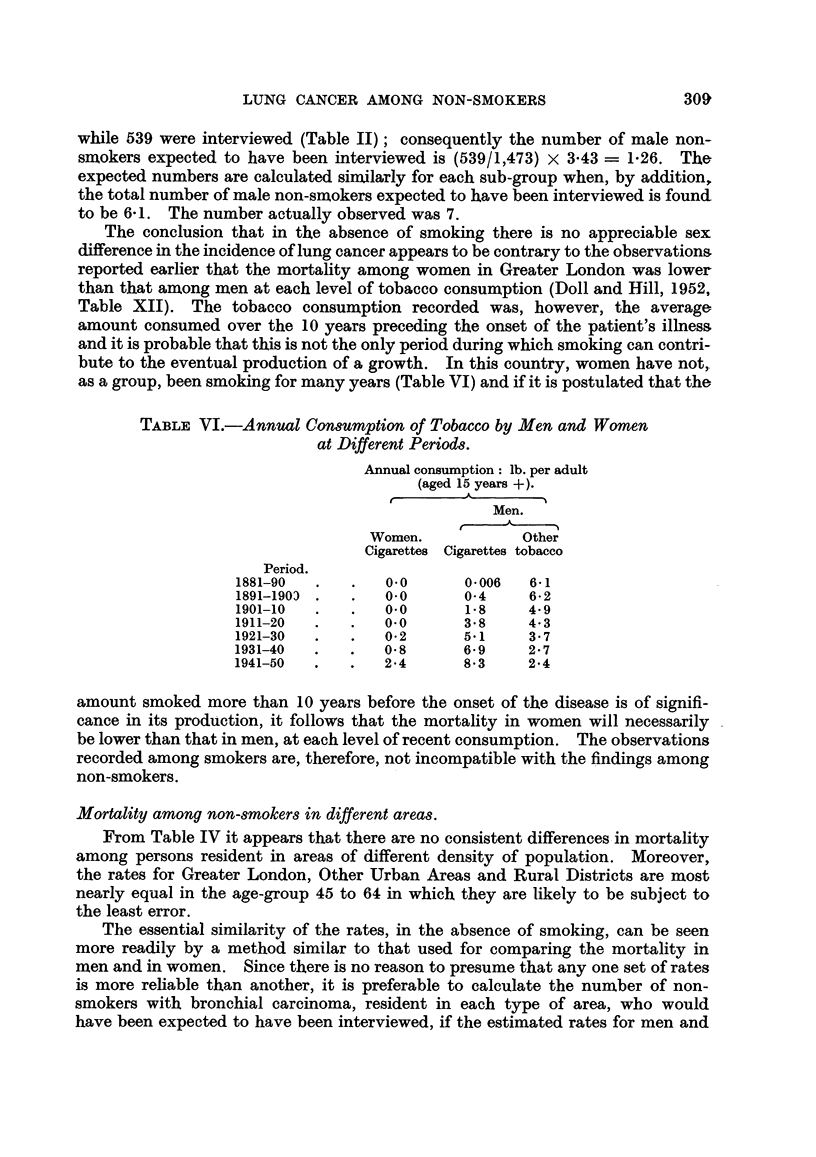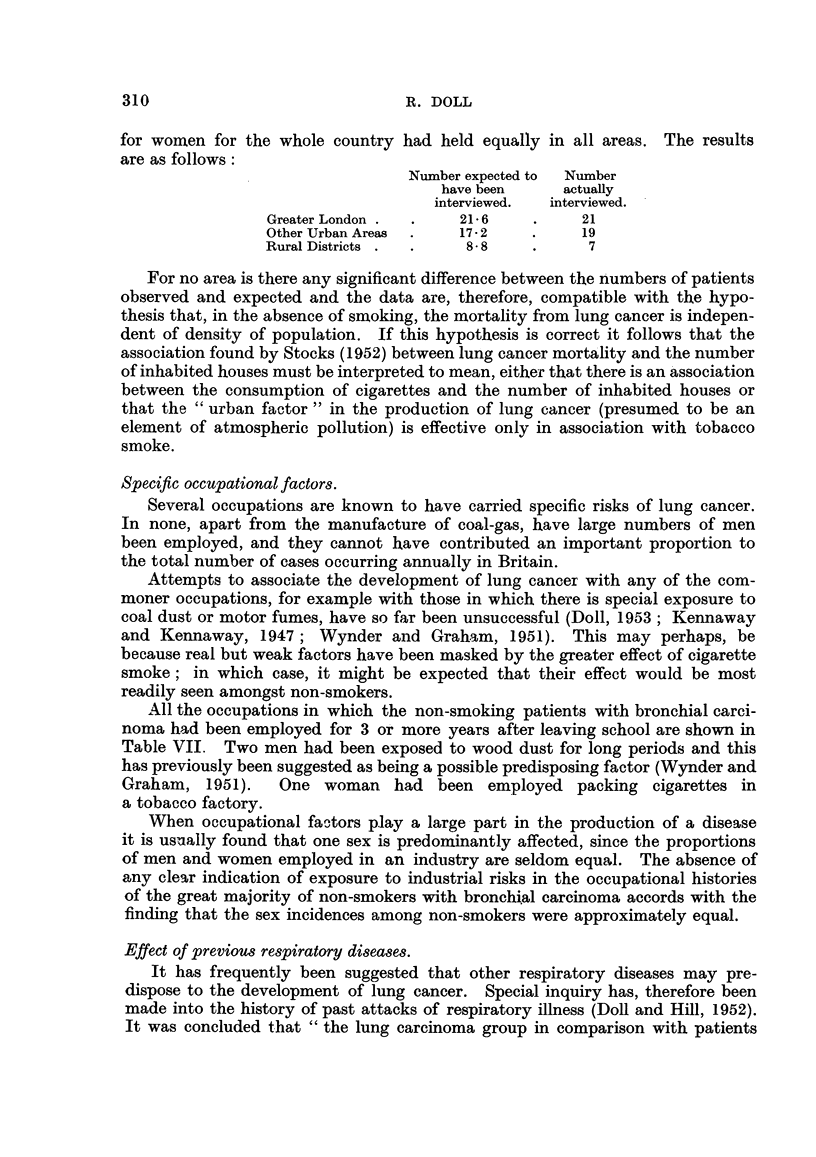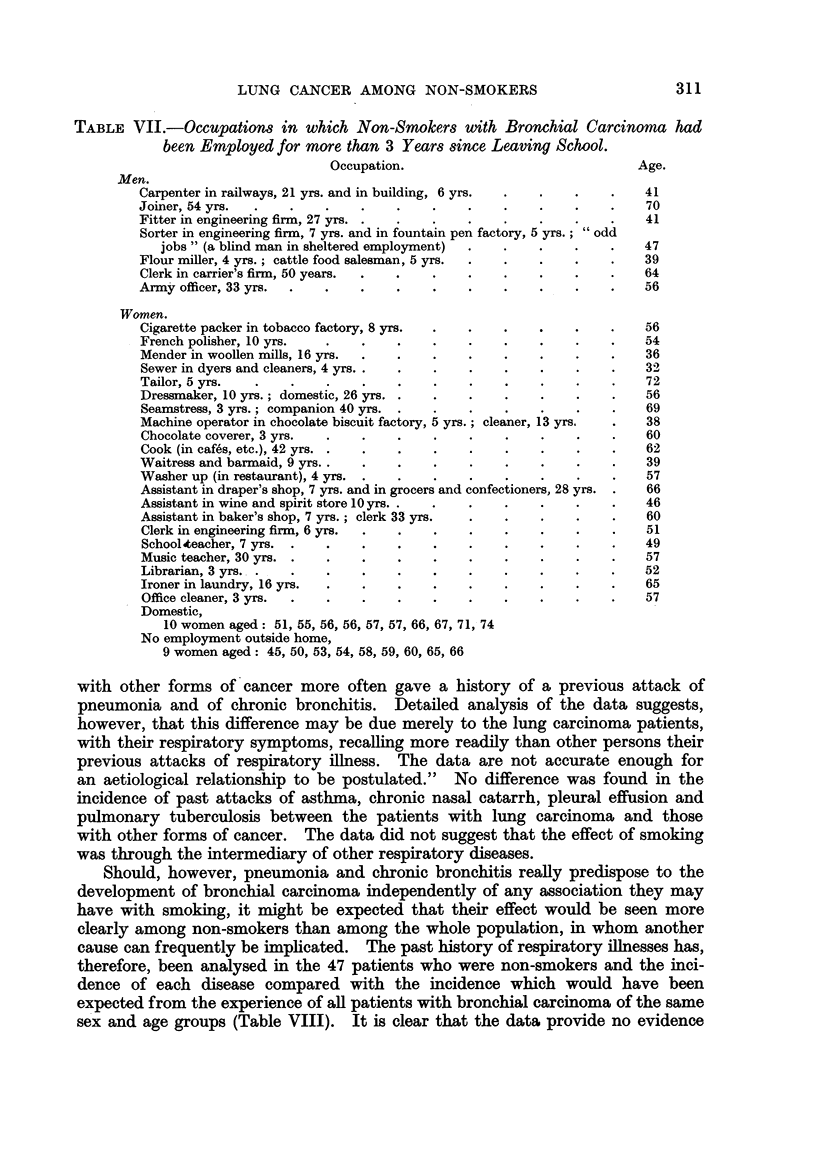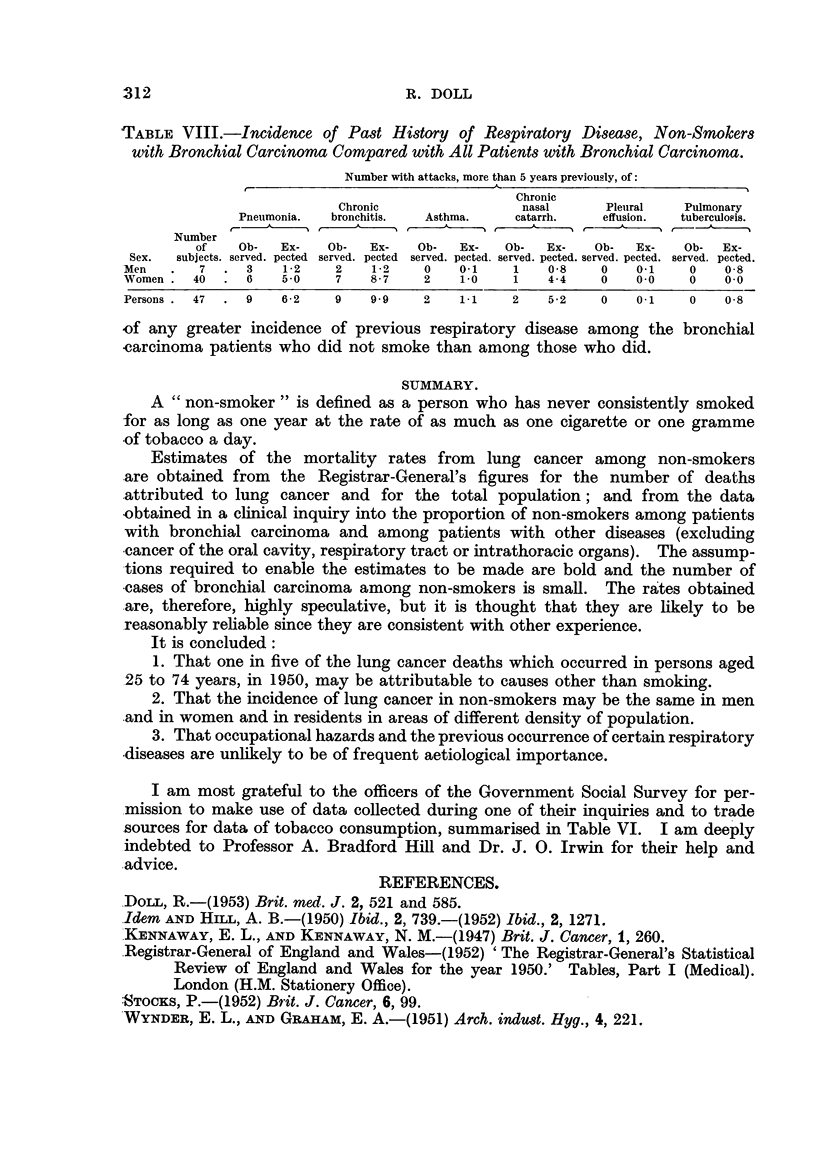# Mortality from Lung Cancer among Non-smokers

**DOI:** 10.1038/bjc.1953.29

**Published:** 1953-09

**Authors:** R. Doll


### MORTALITY FROM LUNG CANCER AMONG NON-SMOKERS.

R. DOLL.

*From the Statistical Research Unit of the Medical Research Council.*

London School of Hygiene and Tropical Medicine, Keppel Street, W.C. 1.

Received for publication July 16, 1953.

ALL reports agree that cancer of the lung occurs among " non-smokers." The definition of a " non-smoker " has, however, varied and has never been so strict as to exclude persons who have smoked only one cigarette, one cigar or one pipe of tobacco. Such a definition would have little interest in England where its use would probably result in no men at all being classified as non-smokers. It would not in any case, delimit a class of persons who had never been exposed to tobacco smoke, since persons who do not themselves smoke, breathe air containing smoke produced by others. Such a rigorous definition is, moreover, probably unnecessary. Even very heavy smoking does not appear to be uniformly carcinogenic and it is therefore unlikely that an appreciable risk should be incurred by smoking on a single occasion.

The definition used by Doll and Hill (1952) is that of a person who has never consistently smoked for as long as one year at the rate of as much as one cigarette or one gramme of tobacco a day. In practice, there is seldom difficulty in deciding whether a person has satisfied this criterion and it is thought to define a qualitatively distinct group since persons who have smoked more than this amount have, with few exceptions, smoked much more. In this sense, some 5 per cent of men and some 50–70 per cent of women are, in England, classified as " non-smokers."

To calculate the lung cancer death rate among non-smokers, it is necessary to estimate two sets of figures : the numbers of non-smokers at risk and the numbers of non-smokers, in corresponding sex and age groups, dying of lung cancer over a given period. The number of non-smokers in the population may be estimated from the figures given by the Registrar-General of England and Wales (1952) and from the data collected by Doll and Hill (1952) from interviews with nearly 5,000 hospital patients in widely separated parts of England.

Table I shows the numbers of men and women, at different ages between the limits of 25 and 74 years, resident in Greater London, Other Urban Areas (County Boroughs and Urban Districts) and Rural Districts at June 30th, 1950 (Registrar-General of England and Wales, 1952). It also shows the numbers of patients with diseases other than cancer of the oral cavity respiratory tract and intrathoracic organs, who fell into the same categories, and the numbers of such patients who were non-smokers. Cancers of the oral cavity, respiratory tract and intrathoracic organs have either been shown to be related to smoking or been suspected of having such a relationship, and patients with cancer at these sites have, therefore, been excluded. In making the estimates here presented, it is assumed that the proportion of non-smokers among the remaining patients with other diseases who were interviewed, was characteristic of that in the total population for each sex, age and place of residence sub-group.

TABLE I.—*Population of England and Wales, Number of Patients* Interviewed and Number of Non-Smokers among them, Subdivided by Sex, Age and Area of Residence.*

| | | Number of men. Area of residence. | | | Number of women. Area of residence. | | |
|---|---|---|---|---|---|---|---|
| Class of subjects. | Age (years). | Greater London. | Other urban areas. | Rural districts. | Greater London. | Other urban areas. | Rural districts. |
| Population at June 30, 1950 | 25– | 1,349,000 | 4,000,000 | 1,220,000 | 1,399,000 | 4,093,000 | 1,159,000 |
| | 45– | 937,000 | 3,006,000 | 917,000 | 1,107,000 | 3,507,000 | 987,000 |
| | 65–74 | 241,000 | 852,000 | 287,000 | 343,000 | 1,158,000 | 340,000 |
| Patients* interviewed | 25– | 169 | 137 | 83 | 63 | 67 | 53 |
| | 45– | 934 | 466 | 190 | 255 | 125 | 63 |
| | 63–74 | 290 | 76 | 54 | 138 | 60 | 26 |
| Non-smokers among patients | 25– | 10 | 17 | 11 | 33 | 38 | 30 |
| | 45– | 38 | 18 | 14 | 155 | 89 | 45 |
| | 65–74 | 15 | 4 | 5 | 102 | 50 | 22 |

* Excluding patients with cancer of the oral cavity, respiratory tract or intrathoracic cavity and those with secondary cancer in whom the site of origin of the primary was uncertain.

The numbers of non-smokers in the population are estimated by multiplying the population in each sub-group by the proportion of non-smokers in the corresponding sub-group of patients. For example, the number of male non-smokers, aged 45–64, living in Greater London at June 30, 1950, is estimated to have been (to the nearest 100)

$$\frac{38}{934} \times 937{,}000 = 38{,}100.$$

The number of non-smokers dying of lung-cancer is estimated in a similar way from the Registrar-General's mortality data (Registrar-General of England and Wales, 1952) and from the proportions of non-smokers found among the group of patients with bronchial carcinoma who were interviewed. The data are shown in Table II.

The estimated numbers of non-smokers living in mid-1950 and the estimated numbers dying of lung cancer in 1950 are shown in Table III. From the data, annual death rates may be calculated separately for each sub-group, or for persons in each type of area (by adding the figures for men and for women), or for men and for women generally (by adding the figures for each type of area). For example, the estimated annual death rate from lung cancer among non-smokers aged 45–64 in Greater London in 1950 is

$$\frac{5{\cdot}5 + 60{\cdot}6}{38{,}100 + 673{,}000} = \frac{66{\cdot}1}{711{,}100} = 0{\cdot}093 \text{ per } 1{,}000,$$

and the estimated annual death rate among men aged 45 to 64, throughout England and Wales is

$$\frac{5{\cdot}5 + 10{\cdot}1 + 0{\cdot}0}{38{,}100 + 116{,}100 + 67{,}600} = \frac{15{\cdot}6}{221{,}800} = 0{\cdot}070 \text{ per } 1{,}000.$$

TABLE II.—*Deaths from Lung Cancer in England and Wales, Number of Patients with Bronchial Carcinoma Interviewed and Number of Non-Smokers among them, Subdivided by Sex, Age and Area of Residence.*

| | | Number of men. Place of residence. | | | Number of women. Place of residence. | | |
|---|---|---|---|---|---|---|---|
| Class of subjects. | Age (years). | Greater London. | Other urban areas. | Rural districts. | Greater London. | Other urban areas. | Rural districts. |
| Persons dying of lung cancer in 1950 | 25– | 170 | 398 | 85 | 39 | 116 | 14 |
| | 45– | 1473 | 3800 | 786 | 215 | 533 | 118 |
| | 65–74 | 752 | 1709 | 334 | 151 | 378 | 98 |
| Patients with bronchial carcinoma interviewed | 25– | 61 | 52 | 14 | 9 | 7 | 0 |
| | 45– | 539 | 376 | 111 | 39 | 23 | 10 |
| | 65–74 | 130 | 40 | 16 | 13 | 3 | 2 |
| Non-smokers among patients | 25– | 0 | 3 | 0 | 1 | 3 | 0 |
| | 45– | 2 | 1 | 0 | 11 | 10 | 5 |
| | 65–74 | 0 | 1 | 0 | 7 | 1 | 2 |

TABLE III.—*Estimated Population of Non-Smokers in England and Wales and Estimated Number of Deaths from Lung Cancer among Non-Smokers, Subdivided by Sex, Age and Area of Residence.*

| | | Estimated Number of men. Area of residence. | | | Estimated Number of women. Area of residence. | | |
|---|---|---|---|---|---|---|---|
| Class of subjects. | Age (years). | Greater London. | Other urban areas. | Rural districts. | Greater London. | Other urban areas. | Rural districts. |
| Population of non-smokers at June 30, 1950 | 25– | 79,000 | 496,400 | 161,700 | 732,800 | 2,321,400 | 656,000 |
| | 45– | 38,100 | 116,100 | 67,600 | 673,000 | 2,497,000 | 705,000 |
| | 65–74 | 12,500 | 44,800 | 26,600 | 253,500 | 965,000 | 287,700 |
| Non-smokers dying of lung cancer in 1950 | 25– | 0·0 | 23·0 | 0·0 | 4·3 | 49·7 | —* |
| | 45– | 5·5 | 10·1 | 0·0 | 60·6 | 231·7 | 59·0 |
| | 64–74 | 0·0 | 42·7 | 0·0 | 81·3 | 126·0 | 98·0 |

* Cannot be estimated because no women with bronchial carcinoma who were resident in rural districts were interviewed.

The estimated death rates for persons resident in Greater London, Other Urban Areas and Rural Districts, and for men and women separately and for all persons living in all areas, are shown in Table IV.

*Reliability of the estimated rates.*

Whether the rates may be relied upon depends primarily on whether the assumptions which were made to enable them to be calculated are valid. That is to say, it depends on whether the patients who were interviewed and who were suffering from various diseases other than cancer of certain special sites, were representative, with regard to the proportion of non-smokers among them, of the population in general ; on whether the patients who had bronchial carcinoma were representative of all persons dying of the disease and on whether the Registrar General's mortality data are accurate.

TABLE IV.—*Estimated Annual Death Rate from Lung Cancer per* 1000 *Non-Smokers.*

| Age (years). | Persons resident in : Greater London. | Other urban areas. | Rural districts. | All areas, England and Wales. Men. | Women. | Persons. |
|---|---|---|---|---|---|---|
| 25– | 0·005 | 0·026 | —* | 0·031 | 0·018 | 0·020 |
| 45– | 0·093 | 0·093 | 0·076 | 0·070 | 0·091 | 0·090 |
| 65–74 | 0·306 | 0·167 | 0·312 | 0·509 | 0·203 | 0·219 |

The difference between any pair of rates for subjects of the same age group is, in no instance, as great as twice the standard error of the difference.

* Between 0·000 and 0·017, depending on the proportion of the 14 female patients dying of lung cancer who were non-smokers.

An opportunity to test the first assumption is provided through the courtesy of the Government Social Survey by the use of figures for the proportions of non-smokers in the population, obtained in the course of an independent inquiry. The subjects were interviewed in September 1951 and were a random sample of the whole population ; the definition of a " non-smoker " was the same as that used by Doll and Hill (1952). A minor point of difference was that the lower age limit of the youngest age group was 21 years, and not 25 years. The total number of subjects interviewed between the ages of 21 and 74 was 2,268 ; the numbers in each age and sex group were proportional to the numbers in the whole population and the numbers of men interviewed—particularly in the older age groups—were considerably smaller than the numbers used for the previous calculations. The results obtained by the use of the Government Social Survey figures for the estimation of the numbers of non-smokers living at June 30, 1950, are shown in Table V. They are in no instance grossly different from those shown in Table IV and in many instances the two sets of figures agree closely.

TABLE V.—*Annual Death Rate from Lung Cancer per* 1000 *Non-Smokers using Government Social Survey Figures for Estimation of the Number of Non-Smokers at Risk.*

| Age (years). | Persons resident in : Greater London. | Other urban areas. | Rural districts. | All areas, England and Wales. Men. | Women. | Persons. |
|---|---|---|---|---|---|---|
| 25– | 0·006 | 0·030 | —* | 0·030 | 0·021 | 0·023 |
| 45– | 0·085 | 0·087 | 0·070 | 0·045 | 0·087 | 0· 84 |
| 65–74 | 0·283 | 0·157 | 0·273 | 0·368 | 0·190 | 0·202 |

* Between 0·000 and 0·017, depending on the proportion of the 14 female patients dying of lung cancer who were non-smokers.

With regard to the second assumption, it can only be said that the method of notification, by which patients were obtained for interview, and the reasons for which the interview failed to be completed on a number of occasions, do not suggest that the patients who were interviewed are likely to have been unrepresentative of patients with bronchial carcinoma (Doll and Hill, 1950 and 1952).

Whether the third assumption is justified is a large question, with implications outside the limits of this paper : it has been discussed with regard to lung cancer by Kennaway and Kennaway (1947). Errors of certification affecting the Registrar-General's mortality data certainly occur in individual cases, but the picture of mortality provided is believed to be accurate in broad outline. In so far as there are greater inaccuracies of certification in one or other sex or in one or other part of the country, the main conclusions of the present paper are also erroneous.

That the order of magnitude of the estimated rates is reasonable, may be illustrated by comparing them with the rates recorded by the Registrar-General for men and for women in different areas of the country (Registrar-General of England and Wales, 1952). From Fig. 1 it is seen that the estimated rates for non-smokers are (with one exception) slightly less than the rates for women in rural districts—that is, the lowest recorded by the Registrar-General. The exception, the rate for non-smokers aged 25 to 44, is derived from the experience of the smallest number of bronchial carcinoma patients and is likely to be the least reliable; this rate lies between the lowest and the lowest but one of the Registrar-General's rates. The estimated rates are, therefore, of the order which could be expected if they represented the basic minima independent of variable environmental factors.

*Proportion of deaths due to causes other than smoking.*

If the estimated rates for non-smokers are accepted as appoximately accurate, it is possible to calculate the number of deaths from lung cancer in persons between the ages of 25 and 74 which would have been expected, in the absence of smoking, by multiplying the populations given in Table I by the rates for all persons given in Table IV and adding the results for each age group. The number expected in 1950 would have been 1,875; the number which actually occurred was 11,189. It must be presumed that the other causes of lung cancer continued to operate irrespective of the use of tobacco, and it is, therefore, concluded that about one in five of the lung cancer deaths in persons aged 25 to 74 in 1950, were attributable to causes other than smoking.

*Sex ratio among non-smokers.*

Table IV shows no consistent difference between the estimated rates for men and for women; and in the age-group 45 to 64, for which the rates must be presumed to be the most reliable because derived from interviews with the greatest number of patients, the rates are most nearly equal. The data are, therefore, not grossly inconsistent with the hypothesis that in the absence of smoking there is no appreciable sex difference in the incidence of lung cancer.

The closeness with which the data fit the hypothesis can be seen more readily if the rates for women (derived from greater numbers of non-smokers than the rates for men) are used to calculate the number of non-smokers among men with bronchial carcinoma who would have been expected to have been interviewed, if men and women suffered the same mortality. For example, the mortality rate for women aged 45 to 64, and resident in Greater London, is estimated to be $60{\cdot}6/673{,}000$ (Table III); the estimated number of male non-smokers in the same age and area of residence sub-group is 38,100 (Table III), so that the expected number of male non-smokers dying of lung cancer in 1950 is $(60{\cdot}6/673{,}000) \times 38{,}100 = 3{\cdot}43$. Altogether 1,473 men in this sub-group died of lung cancer,

FIG. 1.—Lung cancer mortality, 1950, in men and women residents in different areas and in non-smokers. ——— Men. – – – Women. ...... Non-smokers (persons).

| Class of persons. | Lung cancer. Mortality per 1000. Age. 25–. | 45–. | 65–74. |
|---|---|---|---|
| *Men* | | | |
| Greater London . . | 0·126 | 1·572 | 3·124 |
| Other urban areas . | 0·095 | 1·264 | 2·006 |
| Rural districts . . | 0·070 | 0·851 | 1·164 |
| *Women* | | | |
| Greater London . . | 0·028 | 0·194 | 0·440 |
| Other urban areas . | 0·028 | 0·152 | 0·326 |
| Rural districts . . | 0·012 | 0·120 | 0·288 |
| *Non-smokers* (persons) | | | |
| All areas . . . | 0·020 | 0·090 | 0·219 |

while 539 were interviewed (Table II); consequently the number of male non-smokers expected to have been interviewed is (539/1,473) × 3·43 = 1·26. The expected numbers are calculated similarly for each sub-group when, by addition, the total number of male non-smokers expected to have been interviewed is found to be 6·1. The number actually observed was 7.

The conclusion that in the absence of smoking there is no appreciable sex difference in the incidence of lung cancer appears to be contrary to the observations reported earlier that the mortality among women in Greater London was lower than that among men at each level of tobacco consumption (Doll and Hill, 1952, Table XII). The tobacco consumption recorded was, however, the average amount consumed over the 10 years preceding the onset of the patient's illness and it is probable that this is not the only period during which smoking can contribute to the eventual production of a growth. In this country, women have not, as a group, been smoking for many years (Table VI) and if it is postulated that the

TABLE VI.—*Annual Consumption of Tobacco by Men and Women at Different Periods.*

| | Annual consumption: lb. per adult (aged 15 years +). | | |
|---|---|---|---|
| | | Men. | |
| | Women. Cigarettes | Cigarettes | Other tobacco |
| Period. | | | |
| 1881–90 | 0·0 | 0·006 | 6·1 |
| 1891–1900 | 0·0 | 0·4 | 6·2 |
| 1901–10 | 0·0 | 1·8 | 4·9 |
| 1911–20 | 0·0 | 3·8 | 4·3 |
| 1921–30 | 0·2 | 5·1 | 3·7 |
| 1931–40 | 0·8 | 6·9 | 2·7 |
| 1941–50 | 2·4 | 8·3 | 2·4 |

amount smoked more than 10 years before the onset of the disease is of significance in its production, it follows that the mortality in women will necessarily be lower than that in men, at each level of recent consumption. The observations recorded among smokers are, therefore, not incompatible with the findings among non-smokers.

*Mortality among non-smokers in different areas.*

From Table IV it appears that there are no consistent differences in mortality among persons resident in areas of different density of population. Moreover, the rates for Greater London, Other Urban Areas and Rural Districts are most nearly equal in the age-group 45 to 64 in which they are likely to be subject to the least error.

The essential similarity of the rates, in the absence of smoking, can be seen more readily by a method similar to that used for comparing the mortality in men and in women. Since there is no reason to presume that any one set of rates is more reliable than another, it is preferable to calculate the number of non-smokers with bronchial carcinoma, resident in each type of area, who would have been expected to have been interviewed, if the estimated rates for men and for women for the whole country had held equally in all areas. The results are as follows :

| | Number expected to have been interviewed. | Number actually interviewed. |
|---|---|---|
| Greater London . . | 21·6 . | 21 |
| Other Urban Areas . | 17·2 . | 19 |
| Rural Districts . . | 8·8 . | 7 |

For no area is there any significant difference between the numbers of patients observed and expected and the data are, therefore, compatible with the hypothesis that, in the absence of smoking, the mortality from lung cancer is independent of density of population. If this hypothesis is correct it follows that the association found by Stocks (1952) between lung cancer mortality and the number of inhabited houses must be interpreted to mean, either that there is an association between the consumption of cigarettes and the number of inhabited houses or that the " urban factor " in the production of lung cancer (presumed to be an element of atmospheric pollution) is effective only in association with tobacco smoke.

*Specific occupational factors.*

Several occupations are known to have carried specific risks of lung cancer. In none, apart from the manufacture of coal-gas, have large numbers of men been employed, and they cannot have contributed an important proportion to the total number of cases occurring annually in Britain.

Attempts to associate the development of lung cancer with any of the commoner occupations, for example with those in which there is special exposure to coal dust or motor fumes, have so far been unsuccessful (Doll, 1953 ; Kennaway and Kennaway, 1947 ; Wynder and Graham, 1951). This may perhaps, be because real but weak factors have been masked by the greater effect of cigarette smoke ; in which case, it might be expected that their effect would be most readily seen amongst non-smokers.

All the occupations in which the non-smoking patients with bronchial carcinoma had been employed for 3 or more years after leaving school are shown in Table VII. Two men had been exposed to wood dust for long periods and this has previously been suggested as being a possible predisposing factor (Wynder and Graham, 1951). One woman had been employed packing cigarettes in a tobacco factory.

When occupational factors play a large part in the production of a disease it is usually found that one sex is predominantly affected, since the proportions of men and women employed in an industry are seldom equal. The absence of any clear indication of exposure to industrial risks in the occupational histories of the great majority of non-smokers with bronchial carcinoma accords with the finding that the sex incidences among non-smokers were approximately equal.

*Effect of previous respiratory diseases.*

It has frequently been suggested that other respiratory diseases may predispose to the development of lung cancer. Special inquiry has, therefore been made into the history of past attacks of respiratory illness (Doll and Hill, 1952). It was concluded that " the lung carcinoma group in comparison with patients

TABLE VII.—*Occupations in which Non-Smokers with Bronchial Carcinoma had been Employed for more than 3 Years since Leaving School.*

| Occupation. | Age. |
|---|---|
| *Men.* | |
| Carpenter in railways, 21 yrs. and in building, 6 yrs. | 41 |
| Joiner, 54 yrs. | 70 |
| Fitter in engineering firm, 27 yrs. | 41 |
| Sorter in engineering firm, 7 yrs. and in fountain pen factory, 5 yrs.; "odd jobs" (a blind man in sheltered employment) | 47 |
| Flour miller, 4 yrs.; cattle food salesman, 5 yrs. | 39 |
| Clerk in carrier's firm, 50 years. | 64 |
| Army officer, 33 yrs. | 56 |
| *Women.* | |
| Cigarette packer in tobacco factory, 8 yrs. | 56 |
| French polisher, 10 yrs. | 54 |
| Mender in woollen mills, 16 yrs. | 36 |
| Sewer in dyers and cleaners, 4 yrs. | 32 |
| Tailor, 5 yrs. | 72 |
| Dressmaker, 10 yrs.; domestic, 26 yrs. | 56 |
| Seamstress, 3 yrs.; companion 40 yrs. | 69 |
| Machine operator in chocolate biscuit factory, 5 yrs.; cleaner, 13 yrs. | 38 |
| Chocolate coverer, 3 yrs. | 60 |
| Cook (in cafés, etc.), 42 yrs. | 62 |
| Waitress and barmaid, 9 yrs. | 39 |
| Washer up (in restaurant), 4 yrs. | 57 |
| Assistant in draper's shop, 7 yrs. and in grocers and confectioners, 28 yrs. | 66 |
| Assistant in wine and spirit store 10 yrs. | 46 |
| Assistant in baker's shop, 7 yrs.; clerk 33 yrs. | 60 |
| Clerk in engineering firm, 6 yrs. | 51 |
| School teacher, 7 yrs. | 49 |
| Music teacher, 30 yrs. | 57 |
| Librarian, 3 yrs. | 52 |
| Ironer in laundry, 16 yrs. | 65 |
| Office cleaner, 3 yrs. | 57 |
| Domestic, 10 women aged: 51, 55, 56, 56, 57, 57, 66, 67, 71, 74 | |
| No employment outside home, 9 women aged: 45, 50, 53, 54, 58, 59, 60, 65, 66 | |

with other forms of cancer more often gave a history of a previous attack of pneumonia and of chronic bronchitis. Detailed analysis of the data suggests, however, that this difference may be due merely to the lung carcinoma patients, with their respiratory symptoms, recalling more readily than other persons their previous attacks of respiratory illness. The data are not accurate enough for an aetiological relationship to be postulated." No difference was found in the incidence of past attacks of asthma, chronic nasal catarrh, pleural effusion and pulmonary tuberculosis between the patients with lung carcinoma and those with other forms of cancer. The data did not suggest that the effect of smoking was through the intermediary of other respiratory diseases.

Should, however, pneumonia and chronic bronchitis really predispose to the development of bronchial carcinoma independently of any association they may have with smoking, it might be expected that their effect would be seen more clearly among non-smokers than among the whole population, in whom another cause can frequently be implicated. The past history of respiratory illnesses has, therefore, been analysed in the 47 patients who were non-smokers and the incidence of each disease compared with the incidence which would have been expected from the experience of all patients with bronchial carcinoma of the same sex and age groups (Table VIII). It is clear that the data provide no evidence

TABLE VIII.—*Incidence of Past History of Respiratory Disease, Non-Smokers with Bronchial Carcinoma Compared with All Patients with Bronchial Carcinoma.*

| | | Number with attacks, more than 5 years previously, of: | | | | | | | | | | | |
|---|---|---|---|---|---|---|---|---|---|---|---|---|---|
| | | Pneumonia. | | Chronic bronchitis. | | Asthma. | | Chronic nasal catarrh. | | Pleural effusion. | | Pulmonary tuberculosis. | |
| Sex. | Number of subjects. | Ob-served. | Ex-pected | Ob-served. | Ex-pected | Ob-served. | Ex-pected. | Ob-served. | Ex-pected. | Ob-served. | Ex-pected. | Ob-served. | Ex-pected. |
| Men . | 7 . | 3 | 1·2 | 2 | 1·2 | 0 | 0·1 | 1 | 0·8 | 0 | 0·1 | 0 | 0·8 |
| Women . | 40 . | 6 | 5·0 | 7 | 8·7 | 2 | 1·0 | 1 | 4·4 | 0 | 0·0 | 0 | 0·0 |
| Persons . | 47 . | 9 | 6·2 | 9 | 9·9 | 2 | 1·1 | 2 | 5·2 | 0 | 0·1 | 0 | 0·8 |

of any greater incidence of previous respiratory disease among the bronchial carcinoma patients who did not smoke than among those who did.

### SUMMARY.

A " non-smoker " is defined as a person who has never consistently smoked for as long as one year at the rate of as much as one cigarette or one gramme of tobacco a day.

Estimates of the mortality rates from lung cancer among non-smokers are obtained from the Registrar-General's figures for the number of deaths attributed to lung cancer and for the total population; and from the data obtained in a clinical inquiry into the proportion of non-smokers among patients with bronchial carcinoma and among patients with other diseases (excluding cancer of the oral cavity, respiratory tract or intrathoracic organs). The assumptions required to enable the estimates to be made are bold and the number of cases of bronchial carcinoma among non-smokers is small. The rates obtained are, therefore, highly speculative, but it is thought that they are likely to be reasonably reliable since they are consistent with other experience.

It is concluded:

1. That one in five of the lung cancer deaths which occurred in persons aged 25 to 74 years, in 1950, may be attributable to causes other than smoking.
2. That the incidence of lung cancer in non-smokers may be the same in men and in women and in residents in areas of different density of population.
3. That occupational hazards and the previous occurrence of certain respiratory diseases are unlikely to be of frequent aetiological importance.

I am most grateful to the officers of the Government Social Survey for permission to make use of data collected during one of their inquiries and to trade sources for data of tobacco consumption, summarised in Table VI. I am deeply indebted to Professor A. Bradford Hill and Dr. J. O. Irwin for their help and advice.

### REFERENCES.

DOLL, R.—(1953) *Brit. med. J.* **2**, 521 and 585.

*Idem* AND HILL, A. B.—(1950) *Ibid.*, **2**, 739.—(1952) *Ibid.*, **2**, 1271.

KENNAWAY, E. L., AND KENNAWAY, N. M.—(1947) *Brit. J. Cancer*, **1**, 260.

Registrar-General of England and Wales—(1952) 'The Registrar-General's Statistical Review of England and Wales for the year 1950.' Tables, Part I (Medical). London (H.M. Stationery Office).

STOCKS, P.—(1952) *Brit. J. Cancer*, **6**, 99.

WYNDER, E. L., AND GRAHAM, E. A.—(1951) *Arch. indust. Hyg.*, **4**, 221.